# Venom of the Red-Bellied Black Snake *Pseudechis porphyriacus* Shows Immunosuppressive Potential

**DOI:** 10.3390/toxins12110674

**Published:** 2020-10-26

**Authors:** Rachael Y. M. Ryan, Viviana P. Lutzky, Volker Herzig, Taylor B. Smallwood, Jeremy Potriquet, Yide Wong, Paul Masci, Martin F. Lavin, Glenn F. King, J. Alejandro Lopez, Maria P. Ikonomopoulou, John J. Miles

**Affiliations:** 1 Australian Institute of Tropical Health and Medicine, James Cook University, Cairns, QLD 4878, Australia; yide.wong@jcu.edu.au; 2 Centre for Molecular Therapeutics, James Cook University, Cairns, QLD 4870, Australia; 3 School of Environment and Sciences, Griffith University, Nathan, QLD 4111, Australia; a.lopez@griffith.edu.au; 4 QIMR Berghofer Medical Research Institute, Herston, QLD 4006, Australia; Viviana.Lutzky@qimrberghofer.edu.au (V.P.L.); taylor.smallwood@uqconnect.edu.au (T.B.S.); maria.ikonomopoulou@imdea.org (M.P.I.); 5 Institute for Molecular Bioscience, The University of Queensland, St Lucia, QLD 4072, Australia; vherzig@usc.edu.au (V.H.); glenn.king@imb.uq.edu.au (G.F.K.); 6 GeneCology Research Centre, School of Science, Technology and Engineering, University of the Sunshine Coast, Sippy Downs, QLD 4556, Australia; 7 School of Biomedical Sciences, The University of Queensland, St Lucia, QLD 4072, Australia; 8 AB SCIEX, Herston, QLD 4006, Australia; Jeremy.Potriquet@sciex.com; 9 Centre for Tropical Bioinformatics and Molecular Biology, James Cook University, Cairns, QLD 4878, Australia; 10 Translational Research Institute, Brisbane, QLD 4102, Australia; p.masci@uq.edu.au; 11 Centre for Clinical Research, The University of Queensland, Brisbane, QLD 4029, Australia; m.lavin@uq.edu.au; 12 Madrid Institute for Advanced Studies (IMDEA) in Food, CEI UAM+CSIC, 28049 Madrid, Spain

**Keywords:** *Pseudechis porphyriacus*, red-bellied black snake, snake venom, immunosuppression, TNF, CD4^+^ T cells

## Abstract

Venoms act with remarkable specificity upon a broad diversity of physiological targets. Venoms are composed of proteins, peptides, and small molecules, providing the foundation for the development of novel therapeutics. This study assessed the effect of venom from the red-bellied black snake (*Pseudechis porphyriacus*) on human primary leukocytes using bead-based flow cytometry, mixed lymphocyte reaction, and cell viability assays. We show that venom treatment had a significant immunosuppressive effect, inhibiting the secretion of interleukin (IL)-2 and tumor necrosis factor (TNF) from purified human T cells by 90% or greater following stimulation with mitogen (phorbol 12-myristate 13-acetate and ionomycin) or via cluster of differentiation (CD) receptors, CD3/CD28. In contrast, venom treatment did not inhibit TNF or IL-6 release from antigen-presenting cells stimulated with lipopolysaccharide. The reduced cytokine release from T cells was not associated with inhibition of T cell proliferation or reduction of cell viability, consistent with an anti-inflammatory mechanism unrelated to the cell cycle. Deconvolution of the venom using reverse-phase HPLC identified four fractions responsible for the observed immunosuppressive activity. These data suggest that compounds from *P. porphyriacus* venom may be potential drug leads for T cell-associated conditions such as graft versus host disease, rheumatoid arthritis, and inflammatory bowel disease.

## 1. Introduction

Captopril, the first commercially licensed venom-derived drug, was developed in 1981 from a peptide found in venom of the pit viper *Bothrops jararaca* [1]. Five other venom-derived drugs, from snakes, leech, cone snail, and lizard, have subsequently received U.S. Food and Drug Administration (FDA) approval, with others at various stages of clinical and preclinical testing [2,3,4]. While recent reviews have highlighted some common challenges associated with venom-drug discovery [5,6], several promising leads appear to work via immune-mediated mechanisms [7,8]. Among snakes, venom from the South American rattlesnake *Crotalus durissus terrificus* and the Chinese cobra *Naja naja atra* have received considerable attention for their immune-modulating activity, whereas venoms from Australian snakes have not been investigated.

*C. durissus terrificus* causes significant mortality across South America, yet components of its venom have been extensively researched for potential therapeutic applications [9,10,11,12]. In vitro studies have shown that the venom’s principal neurotoxin, crotoxin (CTX), and its isolated basic phospholipase A_2_ (PLA_2_) subunit, downmodulate the expression of surface molecules with crucial stimulatory function on immune cells. Specifically, CTX downmodulates major histocompatibility complex (MHC)-II, cluster of differentiation (CD)40, CD80, and CD86 [12]. CTX also inhibits phosphorylation of the signalling molecules, extracellular signal-regulated kinase (ERK)1/2 and p38-mitogen-activated protein kinase (MAPK), on lipopolysaccharide (LPS)-stimulated bone marrow-derived dendritic cells (BMDCs). Due to inhibited LPS signalling pathways, CTX prevents the secretion of proinflammatory cytokines, such as interleukin (IL)-6, IL-12, and tumor necrosis factor (TNF), from BMDCs [12]. Consequently, T cell proliferation and IL-2 production show significant suppression when determined by mixed lymphocyte reaction (MLR) [12]. 

*C. durissus terrificus* venom and derived toxins have also exhibited anti-inflammatory effects in vivo. Mice pretreated with whole venom or intact CTX show diminished production of IgG antibodies against sheep erythrocyte, chicken ovalbumin, and human serum albumin antigens [13]. While this immunosuppressive effect may present a challenge for the production of *Crotalus* antivenom, it may present an opportunity for drug development [14]. Nunes et al. (2010) demonstrated that pretreatment with whole venom or isolated CTX reduced carrageenan-induced paw edema and peritoneal cell migration in male Swiss mice [15]. This effect was mediated by CTX-induced activation of formyl peptide receptors, a class of G protein-coupled receptor associated with chemotaxis [15]. In mouse models of 2,4,6-trinitrobenzenesulfonic acid solution (TNBS)-induced colitis, treatment with whole venom or CTX caused a shift in the colonic microenvironment from proinflammatory to anti-inflammatory through the downregulation of IL-1β, IL-6, and TNF [10]. Colonic tissue samples from CTX-treated mice also showed a marked increase in regulatory CD4^+^ FoxP3^+^ T cells (T_reg_), along with increased IL-10 and TGFβ concentrations, in the lamina propria [10]. These studies suggest that snake venom is a rich platform from which novel immunotherapies could be discovered [16]. 

Whole *N. naja atra* venom (NNAV) and its principal α-neurotoxin, a PLA_2_ known as cobrotoxin, have likewise shown in vivo therapeutic effects across multiple models of disease, including Freund’s complete adjuvant (FCA)-induced arthritis, systemic lupus erythematosus (SLE), skin allograft rejection, nephropathy, and acute lung injury [17]. Importantly, in vivo data demonstrated that purified cobrotoxin was safe and efficacious at dosages of 5–45 μg/kg (when administered orally) and 2.5–10 μg/kg (when administered subcutaneously) [17].

In a study of adjuvant-induced arthritis, administration of heat-inactivated NNAV significantly alleviated mechanical hyperalgesia in rats by day 20 of the protocol [18]. Additionally, NNAV reduced serum TNF while increasing serum IL-10 levels by day 28 [18]. Kou et al. (2014) demonstrated that oral administration of NNAV up to 80 µg/kg did not cause mortality or abnormal behaviour in mice after 21 days of treatment, but markedly increased the production of sheep red blood cell (SRBC) antibodies in a dose-dependent manner [19]. In another study, NNAV significantly inhibited the proliferation of CD4^+^ Th17 and CD8^+^ T cell populations, known mediators of pathology in various autoimmune disorders, demonstrating the immunomodulatory potential of the venom [19,20,21]. 

Collectively, previous research has highlighted snake venom as a potential source of immunomodulatory compounds. However, Australian snakes, including the red-bellied black snake (RBB) *Pseudechis porphyriacus*, have thus far received minimal scientific attention in this area. We hypothesised that systematic screening of crude snake venom may uncover new immunosuppressive proteins that could be developed as drug leads or drug scaffolds against T cell-driven autoimmune diseases. Here, we show that RBB venom (RBBV) reduced the secretion of proinflammatory cytokines from primary leukocytes stimulated with phorbol 12-myristate 13-acetate and ionomycin (P/I), but not LPS. Given this curious immunological activity, we investigated the effect of RBBV against T cell activation in order to determine its potential as a modulator of T cell function.

## 2. Results

### 2.1. Venom Effect on Purified CD4^+^ T Cells

**Figure A1:**
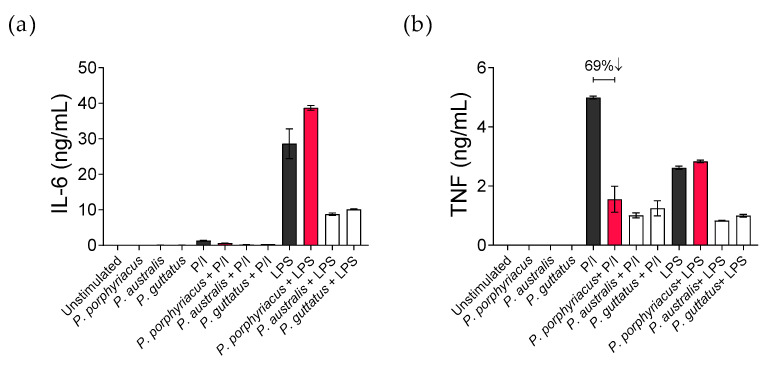
Initial screen of immunosuppressive activity of *Pseudechis* venoms. PBMCs were activated with 50 ng/mL phorbol 12-myristate 13-acetate + 1 µg/mL ionomycin (P/I) or with 100 ng/mL LPS. Activated cells were treated with 1 µg/mL snake venom or remained untreated for 24 h. Secreted (**a**) IL-6 and (**b**) TNF were quantified by CBA. Histograms show the mean cytokine concentration (ng/mL) ± SEM of duplicate samples (*n* = 1 donor). Black bars show the positive mitogen controls (P/I and LPS) while red bars show RBBV-treated samples.

An initial venom screen showed that following 24 h of treatment, RBBV suppressed the secretion of proinflammatory TNF, but not IL-6, from peripheral blood mononuclear cells (PBMCs) stimulated with the mitogen P/I, but not LPS (Figure A1). To investigate the potential of RBBV as an inhibitor of helper T cells, we treated CD4^+^ T cells, isolated from human PBMCs and activated with mitogen (P/I) or T cell receptor (TCR) stimulation (anti-CD3/CD28 beads), with venom for 24 h. The immunosuppressive drug, cyclosporine (CsA), was used as a positive suppressive control.

**Figure 1:**
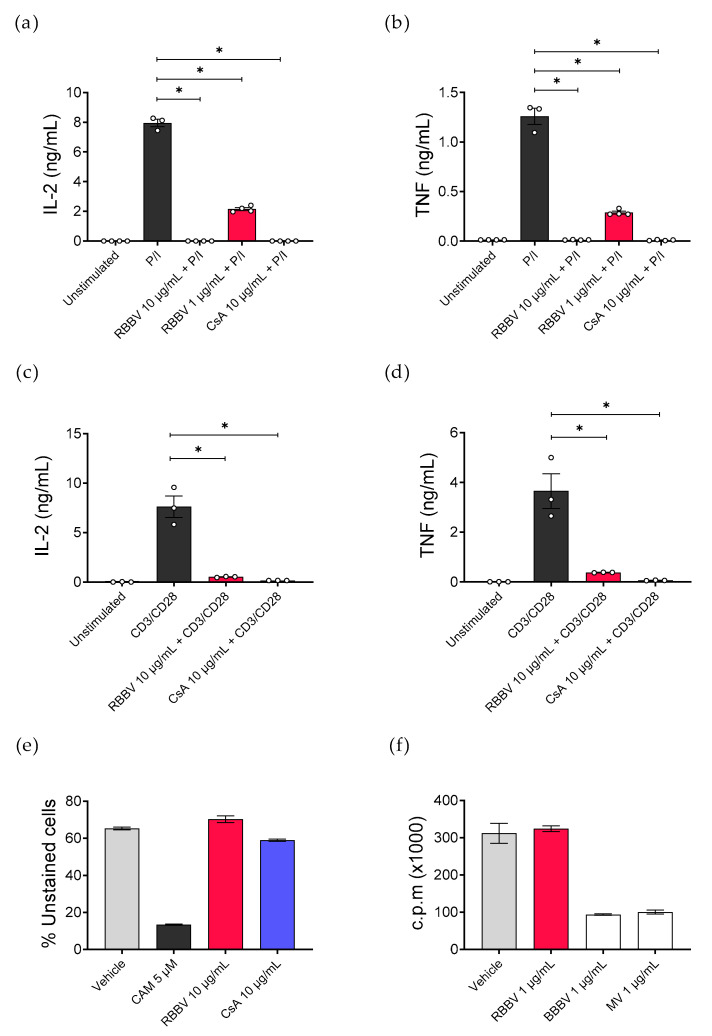
Treatment with red-bellied black snake venom (RBBV) reduces CD4^+^ T cell cytokine secretion without inhibiting cell viability or proliferation. Purified CD4^+^ T cells (*n* = 3 donors) were activated with (**a**,**b**) 50 ng/mL phorbol 12-myristate 13-acetate +1 µg/mL ionomycin (P/I), or with (**c**,**d**) anti-CD3/CD28 stimulation beads at a 1:1 bead to cell ratio. Activated T cells were treated with 1–10 µg/mL RBBV or 10 µg/mL cyclosporine (CsA) for 24 h. Secreted interleukin (IL)-2 and tumor necrosis factor (TNF) were quantified by cytometric bead array (CBA). Histograms show the mean cytokine concentration (ng/mL) ± standard error of mean (SEM) of each experimental group; each point represents the mean of triplicate samples. (**e**) T cell viability. Purified T cells (*n* = 1 donor) were activated with anti-CD3/CD28 stimulation beads then cultured with phosphate-buffered saline (PBS; vehicle control), 5 µM camptothecin (CAM), 10 µg/mL RBBV, or 10 µg/mL CsA for 72 h. Cells were then stained with propidium iodide and the viability determined through the exclusion of positively stained populations. Histograms shows the mean percentage of unstained cells (live cells) ± SEM of triplicate samples. (**f**) Mixed lymphocyte reaction (*n* = 1 donor). Purified T cells (1 × 10^5^ cells per well) and LPS-matured dendritic cells (1 × 10^4^ cells per well) were cultured for 5 days with 1 µg/mL RBBV (*Pseudechis porphyriacus* venom), BBBV (*Pseudechis guttatus* venom), or MV (*Pseudechis australis* venom). The proliferation was assessed by [^3^H]thymidine incorporation during the final 18 h. Data are expressed as mean counts per min (c.p.m x1000) ± SEM of quadruplicate samples. Statistical significance was calculated using a one-way ANOVA with multiple comparisons of control vs. treatments. For all experiments, *p*-values were adjusted using Dunnett’s method. * = *p* < 0.05; ns = *p* > 0.05.

Treatment with RBBV completely inhibited mitogen-induced IL-2 and TNF secretion and caused a 90% reduction in cytokine secretion following TCR activation (Figure 1a–d), which was equivalent to the suppression achieved with CsA. The immunomodulatory effect was dose-dependent. Potent activity was still observed at a dose as low as 1 µg/mL RBBV, with a 73 and 77% reduction in IL-2 and TNF, respectively (Figure 1a,b).

To assess whether RBBV had a direct cytotoxic effect on the cells, we cultured anti-CD3/CD28 activated CD4^+^ T cells for 72 h with venom, and the effect on cell viability was assessed by exclusion of propidium iodide. Camptothecin (CAM), which was used as a positive cytotoxic control, caused 80% toxicity at 5 µM (Figure 1e). In contrast, we observed no significant reduction in cell viability in the venom-treated or CsA-treated groups, compared to the phosphate-buffered saline (PBS; vehicle) control (Figure 1e), demonstrating that the immunosuppression associated with the venom treatment was not a consequence of reduced T cell viability.

Interestingly, despite the significant venom-associated reduction of IL-2 secretion, RBBV treatment (at 1 µg/mL) did not inhibit T cell proliferation in an MLR (Figure 1f). Conversely, 1 µg/mL of venom, from two additional *Pseudechis* species, *P. guttatus* (BBBV), and *P. australis* (MV), suppressed proliferation by 68 and 66%, respectively, emphasising the unique immunomodulatory capacity of RBBV.

### 2.2. Fractionation and Fraction Screen of RBBV

**Figure 2:**
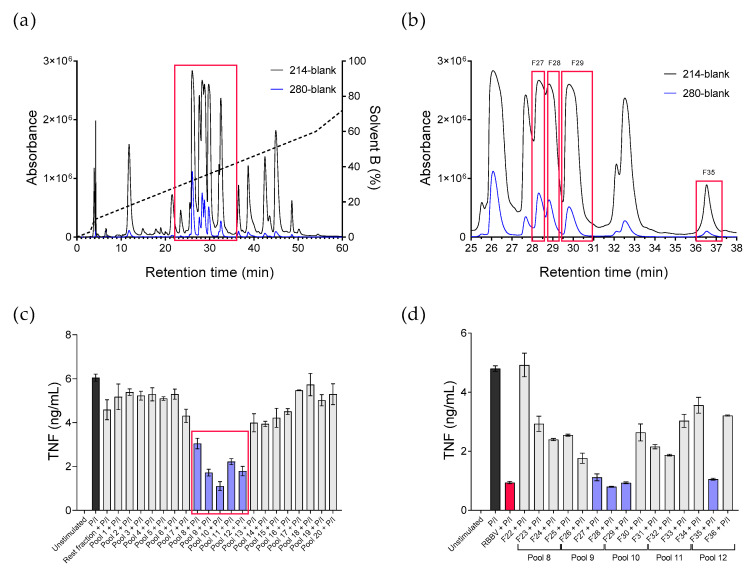
Identification of immunosuppressive RBBV fractions. Reverse-phase high-performance liquid chromatography (RP-HPLC) chromatograms showing fractionation profile of 2 mg RBBV (**a**) and retention times of immunosuppressive fractions in red boxes (**b**). Venom peptide bonds were monitored at 214 nm, and aromatic structures were monitored at 280 nm. Venom fractions were manually collected until 60 min (59 fractions in total). Peripheral blood mononuclear cells (PBMCs) (*n* = 1 donor) were activated with mitogen (50 ng/mL phorbol 12-myristate 13-acetate + 1 µg/mL ionomycin, P/I) and treated with 10 µg/mL RBBV, 120 µg/mL of pooled fractions (3 fractions per pool), or 100 µg/mL of individual venom fraction for 24 h. Secreted TNF was quantified by CBA (**c**,**d**). Histograms show the mean cytokine concentration (ng/mL) ± SEM of triplicate samples.

To identify the immunosuppressive compound/s in RBBV, we fractionated 2 mg venom by reverse-phase high-performance liquid chromatography (RP-HPLC) (Figure 2a). Fifty-nine fractions were collected, and all were screened for inhibitory effect on mitogen-induced (P/I) TNF secretion. Five fraction pools (pools 8 to 12), containing fractions 22 to 36, suppressed PBMC TNF secretion by 63–82%, compared to the untreated control (Figure 2c). Fractions F27, F28, F29, and F35, which eluted at 28.07, 28.72, 29.56, and 36.03 min, respectively, produced the greatest level of TNF suppression (Figure 2b,d). We observed an 80% inhibition of cytokine secretion in the presence of venom fractions following 24 h of treatment, similar to the immunosuppressive profile of unfractionated RBBV. 

### 2.3. Immunosuppressive and Cytotoxic Activity of Fractions

**Figure 3:**
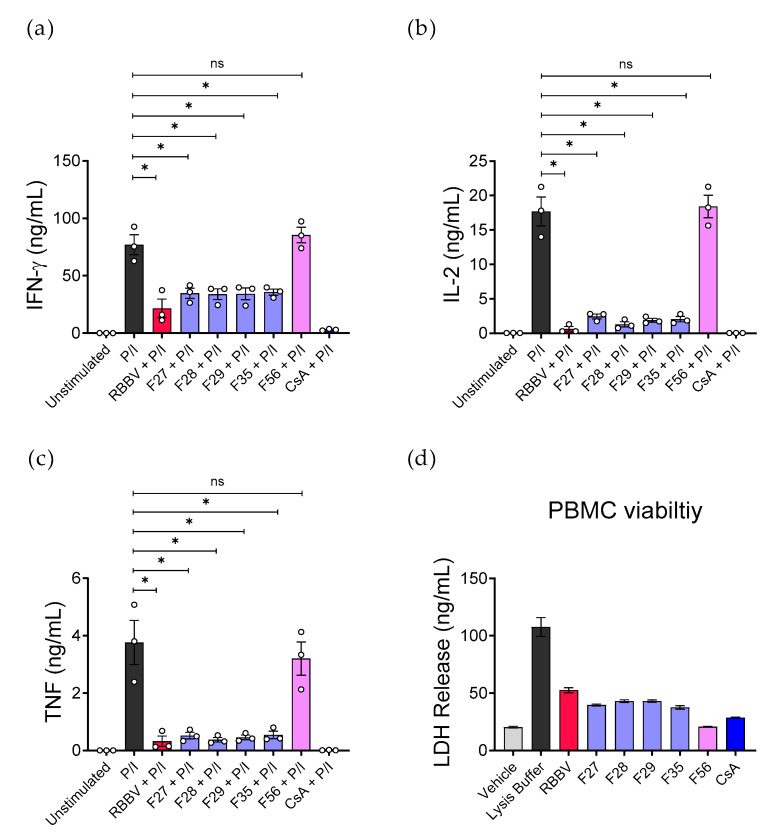
Fractions isolated from RBBV suppressed PBMC cytokine secretion. PBMCs (*n* = 3 donors) were stimulated in triplicate with mitogen (50 ng/mL phorbol 12-myristate 13-acetate + 1 µg/mL ionomycin, P/I) and treated with 10 µg/mL RBBV, 100 µg/mL immunosuppressive fractions (F27, F28, F29, or F35), 100 µg/mL of non-suppressive fraction (F56), or 10 µg/mL CsA for 24 h. Secreted (**a**) IFN-γ, (**b**) IL-2, and (**c**) TNF were quantified by CBA. The cytotoxicity of fractions was assessed by lactate dehydrogenase (LDH) release from PBMCs (*n* = 1 donor) (**d**). Histograms show the mean cytokine concentration (ng/mL) ± SEM of each experimental group; each point represents the mean of triplicate samples. Statistical significance was calculated using a one-way ANOVA with multiple comparisons of control vs. treatment. *p*-values were adjusted using Dunnett’s method. * = *p* < 0.05; ns = *p* > 0.05.

Next, the activity of the immunosuppressive fractions was evaluated against the release of mitogen-induced (P/I) Th1-type cytokines from PBMCs obtained from multiple donors (Figure 3a–c). We observed that in addition to 86% inhibition of TNF secretion, all four fractions suppressed the secretion of interferon gamma (IFN-γ) and IL-2 by 55% and 89%, respectively. Again, the cytokine inhibition produced by these fractions was similar to unfractionated RBBV. Conversely, fraction 56 (F56), which eluted at the highest concentration of solvent B and was included as a negative control, had no significant effect on cytokine secretion. 

To assess the cytotoxicity of the bioactive fractions, we measured the release of cytosolic lactate dehydrogenase (LDH) into PBMC culture supernatant following 24 h of treatment (Figure 3d). We observed an increase in LDH secretion from cells treated with RBBV (2.6×); fractions F27, F28, F29, and F35 (2×); and CsA (1.4×), but not F56, compared to the untreated cells. This result suggests slight disruption to the integrity of the cell membrane which was likely dependent on the compound/s and concentration in the fraction.

### 2.4. Mass Spectrometry (MS) Analyses

Finally, to further define the immunomodulatory components, we separated each of the four cytokine-suppressive fractions into 24 bands on a one-dimensional (1DE) SDS-PAGE gel. For fractions F27, F28, and F29, we pooled gel bands into three subfractions while the gel bands from fraction F35 were combined into a single pool. The pooled bands were trypsinised, processed, and acquired with an information-dependent acquisition (IDA) method on a mass spectrometer coupled to a reverse phase chromatography system via a nano-electrospray ion source. The acquired spectra were then identified with The Paragon Algorithm against a library of publicly available proteins tagged as toxins (Table A1, Table A2, Table A3, Table A4, Table A5, Table A6, Table A7, Table A8, Table A9 and Table A10). Multiple unique peptide sequences from *P. porphyriacus*-derived pseudexin B were identified in all analysed subfractions. Multiple unique peptide sequences from *P. porphyriacus*-derived pseudexin A were also detected, but not in all subfractions. The non-suppressive fractions were not assessed, however, and therefore we cannot comment on the presence of pseudexin peptides among the non-suppressive fractions. Promisingly, these data are consistent with specific venom components being responsible for the observed anti-inflammatory effect of RBBV. Similar effects have been reported with *C. durissus terrificus*-derived crotoxin and *N. naja. atra*–derived cobrotoxin warranting further investigation of RBBV.

## 3. Discussion

To our knowledge, this study has for the first time identified the immunosuppressive capacity of *P. porphyriacus* venom against activated human lymphocytes. The red-bellied black snake is one of the most commonly encountered venomous species across Eastern Australia [22]. Unlike deadly bites from other *Pseudechis* snakes, such as the mulga snake (*P. australis*), the symptoms of RBB envenomation are typically mild to moderate [23]. Although *P. porphyriacus* venom research dates back to 1930 [24], the majority of the available literature is centred either on the symptoms of envenomation, treatment strategies, or both [25,26,27]. Recently, Goldenburg et al. described the proteomic variation among *Pseudechis* venoms, including *P. porphyriacus,* however, research on RBBV-derived therapeutic leads remains limited [28].

Our work demonstrates that RBBV suppresses the production of T cell cytokines, IL-2, and TNF, by 90% or greater when generated in response to P/I or CD3/CD28 receptor stimulation. Importantly, this occurred without cytotoxic effect. This finding was observed in both purified CD4^+^ T cells and whole PBMCs. We further noted that RBBV cytokine inhibition was equivalent to that of CsA, demonstrating venom potency. As T cells play a pivotal role in the pathogenesis of autoimmune diseases, these data highlight RBBV as a new anti-inflammatory drug lead or drug scaffold [29]. 

Interestingly, our preliminary results showed that RBBV had little effect on LPS-stimulated cytokines. This was supported by our finding that whole venom did not inhibit T cell proliferation in an MLR using allogenic LPS-matured dendritic cells (DC). Such a finding suggests a T cell-specific or T cell regulatory pathway mechanism of action, or a combination of both. However, further research is required to determine the precise receptors and pathways involved. 

To date, limited research has been performed on the therapeutic potential of RBBV-derived toxins, and no literature is currently available regarding its immunomodulatory effects. A study by Bradshaw et al. (2016) showed that RBBV treatment inhibited the proliferation of human breast (MCF-7) and melanoma (A-375) cancer cell lines [30]. At 190 µg/mL RBBV, the authors found growth inhibition of 70–80% when assessed by 3-(4,5-dimethylthiazol-2-yl)-2,5-diphenyltetrazolium bromide (MTT) proliferation assay. Micrograph data confirmed venom cytotoxicity through morphologic changes yet showed minimal effect on cell proliferation at concentrations used in our assays. As previously discussed, experiments by Xu et al. (2015) and others have addressed snake venom toxicity, including that of *N. n. atra,* through heat-induced denaturation [31]. Although our active concentration of 10 µg/mL RBBV was not cytotoxic in vitro, heat inactivation may be required to attenuate unwanted toxicity for future in vivo studies if the non-toxic bioactive molecule cannot be identified and artificially synthesised. 

Pseudexin isoenzymes A, B, and C, were first isolated and characterised from RBBV by Schmidt and Middlebrook in 1989 [32]. The proteins, which are basic PLA_2_ neurotoxins, showed similar specific phospholipase activity to crotoxin. Lethality studies using mice revealed LD_50_ values of 1300 µg/kg, 750 µg/kg, and no apparent symptoms below 8000 µg/kg from intraperitoneal injections of pseudexin A, B, and C, respectively. Preliminary venom fractionation and IDA MS/MS analysis detected multiple unique peptide sequences from *P. porphyriacus*-derived pseudexin B in all analysed subfractions. Multiple unique peptide sequences from *P. porphyriacus*-derived pseudexin A were also detected, but not in all subfractions. Although the presence of pseudexin in our active fractions and its role as the active protein is yet to be validated, several studies have demonstrated an anti-inflammatory effect of certain secretory PLA_2_, such as the already discussed crotoxin [12], as well as those found in bee venom [33] and the group V PLA_2_ isoforms [34]. Accordingly, RBBV-derived PLA_2_ may likewise exhibit anti-inflammatory activity; however, further validation is required. 

Taken together, these data demonstrate the potential for novel medicinal applications of RBBV-derived proteins. Given the extent of T cell cytokine suppression observed in our experiments, we recommend investigating the safety and therapeutic efficacy of RBBV and pure toxins in animal models of disease including the T cell transfer (TcT) model of colitis, FCA-induced arthritis, SLE, or skin allograft rejection. However, it is critically important to isolate, identify, and validate the active component before further therapeutic assessment.

## 4. Materials and Methods 

### 4.1. Human Ethics

The human blood for this research was supplied by the Red Cross under the agreement number 14-11QLD-07 (19/12/2014). Healthy donor blood was studied using protocols carried out in accordance with guidelines and regulations under QIMR Berghofer Medical Research Institute (QIMRB; Brisbane, Australia) (HREC P2058 03/09/2014). All methods and human participants involved in the study were approved by the QIMRB HREC. Informed consent was obtained by the Red Cross from all participants in the study. The study was carried out according to the rules of the Declaration of Helsinki of 1975.

### 4.2. Snake Venom Samples

*P. porphyriacus, P. guttatus*, and *P. australis* venoms were either part of a private collection owned by Dr Paul Masci and Prof. Martin Lavin (The University of Queensland) or purchased from Venom Supplies Pty. Ltd. (Tanunda, SA, Australia). The purchased lyophilised venom was supplied reconstituted at 10 mg/mL in Dulbecco’s phosphate-buffered saline (PBS) (Thermo Fisher Scientific), 50% glycerol. The samples were collected over the course of a year from specimens in Queensland and South Australia, and to reduce seasonal and individual variation, we pooled and lyophilised venom samples from over 40 individual specimens. Venom samples were tested for Gram-negative bacterial contamination using a Limulus amebocyte lysate (LAL) QCL-1000 (Lonza, Morristown, NJ, USA), according to the manufacturer’s instructions.

### 4.3. PBMC Preparation

Cell cultures were maintained in R10 medium containing RPMI-1640 media without L-glutamine (Gibco Thermo Fisher Scientific, Waltham, MA, USA), supplemented with 10% heat-inactivated foetal bovine serum (FBS; Bovogen Biologicals, Christchurch, New Zealand), 10,000 units/mL of penicillin + 10,000 µg/mL of streptomycin (Thermo Fisher Scientific), and 1X GlutaMAX (Thermo Fisher Scientific). 

PBMCs were separated from the buffy coats of three healthy donors by standard density gradient centrifugation using Lymphoprep medium (STEMCELL Technologies, Vancouver, Canada) according to the manufacturer’s instructions. Isolated cells were used fresh or cryopreserved in liquid nitrogen vapour phase storage using 90% heat-inactivated foetal calf serum (FCS) (Bovogen Biologicals) plus 10% dimethyl sulfoxide (DMSO; Sigma-Aldrich, St. Louis, MO, USA). 

On the day of the experiment, PBMCs were thawed and removed from freezing medium by washing with 10 mL R10 and centrifugation at 500 × *g* for 5 min. To reduce cell clumping, we resuspended the pellet in 5 mL R10 with 10 µg/mL DNase solution I (STEMCELL Technologies) and incubated it at 37 °C and 5% CO_2_ for 1 h. The cells were washed twice to remove DNase. Next, PBMCs were counted using a 0.4% trypan blue staining solution (Thermo Fisher Scientific). The cell density was adjusted as required. 

### 4.4. CD4^+^ T Cell Purification.

**Figure A2:**
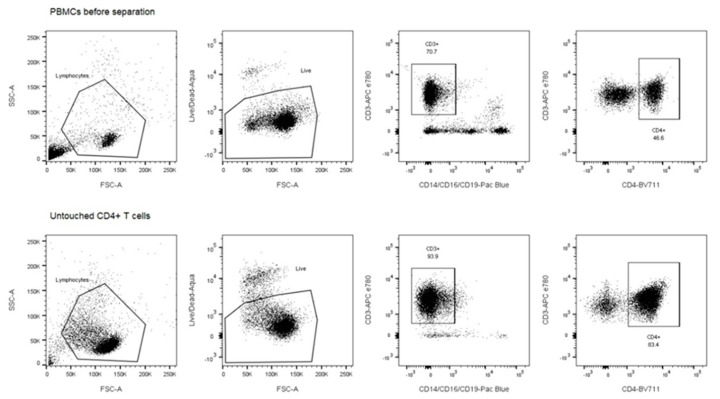
Example gating hierarchy comparing PBMCs before magnetic-activated cell sorting (MACS) to isolated CD4^+^ T cells after separation. The purity of untouched T cells was determined by gating on live lymphocytes after the elimination of doublets and followed by the exclusion of CD14, CD16, and CD19 positive populations.

The isolation of untouched CD4^+^ T cells was performed using magnetic-activated cell sorting (MACS) CD4^+^ T cell isolation kits (Miltenyi Biotec, Cologne, Germany) and MACS LS columns, according to the manufacturer’s instructions. The microbead buffer for MACS separation assays contained 19 parts autoMACS Rinsing Solution and 1 part MACS bovine serum albumin (BSA) Stock Solution for a solution of phosphate-buffered saline (pH 7.2), 0.5% bovine serum albumin, and 2 mM ethylenediaminetetraacetic acid (EDTA). The labelled cells were passed through a 30 µm pre-separation filter before and after separation in order for us to obtain a single-cell suspension. Aliquots of pre-labelled and unlabelled cells were taken for evaluation of purity (Figure A2). The cell preparations with ≥93% CD3^+^ purity as measured by flow cytometry were used for downstream T cell assays.

### 4.5. In Vitro Stimulation

The in vitro activation of immune cells was performed in two ways. Cells were either activated with a cell stimulation cocktail of 50 ng/mL of phorbol 12-myristate 13-acetate and 1 µg/mL of ionomycin (eBioscience Thermo Fisher, San Diego, CA, USA), or Dynabeads Human T-Activator CD3/CD28 beads (Thermo Fisher Scientific) were used according to the manufacturer’s instructions (*n* = 3). Washed Dynabeads were added to the cells at a ratio of 1:1 (bead to cell) by the addition of 25 µL of beads per 1 × 10^6^ T cells. Finally, 2 µL of recombinant IL-2 (R&D systems) per milliliter of culture media was added. For unstimulated control samples, we treated the cells with 5 µL/well PBS, unless otherwise indicated.

Activated T cells were seeded at a density of 140,000 cells/100 µL medium, or unpurified PBMCs were seeded at 100,000 cells/50 µL medium into the wells of 96 well round-bottom plates. The cells were then treated with PBS, 10 µg/mL CsA (Sigma-Aldrich), or 1–10 µg/mL *P. porphyriacus* venom for a final volume of 150 µL or 100 µL for T cells or PBMCs, respectively. To test the activity of *P. porphyriacus* fractions, we used a volume of 1 µL fraction/100 µL media, representing 100 µg/mL venom equivalent. A 10-fold higher venom equivalent concentration of each fraction was used in order to account for potential losses during fractionation and to address potential additive or synergistic effects caused by multiple active toxins present in different venom fractions. The culture plates were incubated at 37 °C and 5% CO_2_ for 24 h. After incubation, the plates were centrifuged at 500× *g* for 5 min and the culture supernatant was collected. Supernatant aliquots were used fresh or were preserved at −80 °C until required.

### 4.6. Propidium Iodide Viability Assays

CD4^+^ T cells, purified and activated with Dynabeads as already described, were treated with PBS, 5 µM CAM, 10 µg/mL *P. porphyriacus* venom, or 10 µg/mL CsA for 72 h (*n* = 1). After incubation, the activation beads were removed using a MACS magnet. The supernatant, containing the cells, was collected and the beads discarded. The samples were washed twice with cold PBS and centrifugation at 500× *g* for 5 min. After a second wash, the cell pellet was resuspended in 1 µL propidium iodide staining solution (BD Biosciences, San Jose, CA, USA) and 49 µL 1X binding buffer, per sample. The stained cells were incubated in the dark, at room temperature, for 15 min. The volume of the wells was adjusted to 200 µL by the addition of 150 µL cold fluorescence-activated cell sorting (FACS) buffer. Finally, the sample data were acquired by flow cytometry using a five laser Special Order LSRFortessa, with a stopping gate of 20,000 events. 

### 4.7. LDH Release Cytotoxicity Assays

PBMC viability, following treatment with RBBV and venom fractions, was determined using a LDH cytotoxicity assay (Promega, Madison, WI, USA). Experiments were performed according to the manufacturer’s instructions. In brief, PBMCs were treated with test compounds or vehicle controls, and the cells cultured at 37 °C and 5% CO_2_ for 24 h. Maximum cell lysis control samples were lysed using 10% Triton X-100 lysis buffer 45 min before cell supernatant collection. Following incubation, culture plates were centrifuged at 500× *g* for 5 min. Culture supernatant was collected, and LDH standards (Sigma-Aldrich) were prepared in duplicate. Samples (25 µL) were mixed with the substrate (25 µL) and incubated for 15 min in the dark at room temperature. To terminate the reaction, we added stop solution (25 µL) to all samples. The plate absorbance was measured at 490 nm using a Gen5 microplate reader (BioTek, Winooski, VT, USA). The LDH sample values, within the linear range of the assay, were converted to ng/mL using the standard curve.

### 4.8. Measurement of Cytokines

Soluble cytokines IFN-γ, IL-2, IL-6, and TNF in cell culture supernatant were quantified using cytometric bead array (CBA) flex sets (BD Biosciences). CBA experiments were performed according to the manufacturer’s instructions with adjusted bead concentrations. Before experiments, frozen supernatants were thawed on ice and then centrifuged at 500× *g* for 5 min to pellet particulates. The CBA samples were acquired according to the manufacturer’s instructions using a 5-laser Special Order LSRFortessa with a high throughput sampler (HTS) (Becton, Dickinson and Company, Franklin Lakes, NJ, USA). Cytokine concentrations (ng/mL) were calculated using the mean fluorescent intensity of sample phycoerythrin (PE) compared to the protein standard curves. BD FCAP Array software version 3.0 was used for data analyses. 

### 4.9. Mixed Lymphocyte Reaction (MLR)

All MLR assays were performed using a previously published method [35]. The PBMCs were isolated by centrifugation of blood through a Ficoll-Paque (GE Healthcare Life Sciences, USA) gradient. Monocytes were then purified from PBMC by positive selection with CD14 microbeads (Miltenyi Biotec) and cultured for 5 days in RPMI 1640 medium with 2 mmol/L-glutamine (Thermo Fisher Scientific), 50 µg/mL gentamicin (Sigma-Aldrich), 800 IU/mL granulocyte-macrophage colony-stimulating factor (Peprotech Inc. Rocky Hill, NJ, USA), 500 IU/mL IL-4 (Miltenyi Biotec), and 10% heat-inactivated FBS. After 5 days, the differentiated dendritic cells were incubated overnight with 1 µg/mL LPS (Sigma-Aldrich) to induce maturation. The purity of monocyte-derived dendritic cells (moDCs) was assessed by immunofluorescence staining with monoclonal antibodies (mAbs) to human leukocyte antigen (HLA)-DR, CD1b, and CD86. The moDC cultures with >97% purity were used for experiments.

Untouched T cells were isolated from PBMCs by RosetteSep immunodensity cell separation (STEMCELL Technologies) and coculture with allogeneic moDCs at a ratio of 10:1. The mixed cultures were treated with glycerol or 1 µg/mL snake venom for a further 5 days and pulsed with [^3^H]thymidine (1µCi/well, specific activity 5 mCi/mmoL; Amersham Life Sciences) for the final 18 h. The mean values ± SEM were plotted using GraphPad Prism version 8.0. 

### 4.10. Reversed-Phase High-Performance Liquid Chromatography

Fractionation of *P. porphyriacus* venom was performed using an analytical reverse-phase Jupiter C18 (250 × 4.6 mm) column (Phenomenex, Sydney, Australia), with a 1 mL injection loop, connected to a Shimadzu Prominence HPLC system (Shimadzu Scientific Instruments, Rydalmere, NSW, Australia). Two milligrams of venom was dissolved in 1 mL of 0.1% trifluoroacetic acid (TFA) in H_2_O. Elution occurred at a flow rate of 1 mL per min according to varying linear gradients of solvent A (0.1% TFA) and solvent B (90% acetonitrile (ACN) + 0.09% TFA). The percentage of solvent B was increased from 0% to 10% over the first 4 min and then from 10% to 80% over 70 min. Venom peptide bonds were monitored at 214 nm, and aromatic structures were monitored at 280 nm. Fifty-nine venom fractions were collected and dried in a centrifugal evaporator. Fractions were then resuspended in 200 µL of PBS, representing a venom equivalent concentration of 10 µg/µL, and stored at −80 °C until required.

### 4.11. Proteomic Analysis of P. porphyriacus Active Fractions (In-Gel Fractionation)

Approximately 24 µg of proteins extracted from the *P. porphyriacus* immunosuppressive fractions were resolved on 12% 1DE SDS-PAGE gels. After gel fixation, the proteins were stained with EZBlue G-250 colloidal Coomassie Stain (Sigma-Aldrich), and each lane was fractionated into 24 gel bands. In-gel trypsin digestion was performed [36], and the mixture of peptides was collected then dried in speed vacuum and frozen at −20 °C.

### 4.12. Mass Spectrometry (MS) Analyses: Information-Dependent Acquisition (IDA)

Peptide fractions from in-gel digest were resuspended in 0.1% TFA and desalted by solid-phase extraction using ZipTip C18 tips, 5 µg (Millipore). Briefly, the C18 tips were washed and activated with 70% ACN/0.1% TFA and then equilibrated with 0.1% TFA before loading the peptides. The wash steps were performed with 0.1% TFA and elution with 80% ACN/0.1% TFA before drying in speed vacuum.

The desalted peptide fractions were resuspended in 20 µL of 0.2% ACN/0.1% TFA and analysed by reverse-phase chromatography with an Eksigent nanoflex cHiPLC coupled to a nano-electrospray TripleTOF 5600 mass spectrometer (AB SCIEX).

Chromatographic separation was achieved on the Eksigent analytical cHiPLC column (3 μm, ChromXP C18CL, 120 Å, 15cm × 200 µm) using three consecutive linear gradients: 5–10% solvent B (ACN/0.1% formic acid) over 2 min; 10–40% solvent B over 58 min; and 40–50% solvent B over 5 min, at a 500 nL/min flow rate.

Peptide spectras were acquired in positive mode using electrospray ionisation (voltage 2300V) using the IDA method. Ions exceeding a threshold of 50 counts and possessing a charge state of +2 to +4 were selected to trigger the acquisition of product ion spectra for the 10 most intense ions with 10 s exclusion after 1 occurrence.

### 4.13. IDA Data Analysis

ProteinPilot v4.5 (AB SCIEX) using The Paragon Algorithm (version 4.5.0.0) was used for the spectral matching analysis against a UniProt combined database consisting of cross-species sequences with biological activity tagged as toxins [37]. 

Background correction was used, and biological modifications specified as an ID focus. The detected protein threshold was set as 0.5, and the false-discovery rate evaluation FDR was set to 1%. 

### 4.14. Statistical Analysis

Statistical analyses were conducted using Prism version 8.0 (Graphpad Software Inc., San Diego, CA, USA). Significance was calculated using a one-way ANOVA with *p*-values adjusted using Dunnett’s method. Significance was defined at *p* ≤ 0.05. Data from histograms are represented as mean ± SEM. 

## Appendix A. Supplementary Data

**Table A1:** Peptides identified in crude RBBV Fraction F27 Pool 1.

Accession	Protein	Hit Species	Expect	Peptide Hits	Total Peptides	Coverage
sp|P20257|PA2BE_PSEAU	Basic phospholipase A2 PA-15 OS = *Pseudechis australis* PE = 1 SV = 1	*Pseudechis australis*	−10.8	2	129	16.9
sp|P20259|PA2BB_PSEPO	Basic phospholipase A2 pseudexin B chain OS = *Pseudechis porphyriacus* PE = 1 SV = 1	*Pseudechis porphyriacus*	−46.9	7	74	68.4
sp|P20255|PA2BF_PSEAU	Basic phospholipase A2 PA-12A OS = *Pseudechis australis* PE = 1 SV = 1	*Pseudechis australis*	−30.1	5	73	31.4
sp|P20258|PA2BA_PSEPO	Basic phospholipase A2 pseudexin A chain OS = *Pseudechis porphyriacus* PE = 1 SV = 1	*Pseudechis porphyriacus*	−30.1	5	72	29.9
sp|P20256|PA2BC_PSEAU	Basic phospholipase A2 PA-12C OS = *Pseudechis australis* PE = 1 SV = 1	*Pseudechis australis*	−21.9	4	63	34.7
sp|Q9PUH7|PA2A7_AUSSU	Acidic phospholipase A2 S15-109 OS = *Austrelaps superbus* PE = 2 SV = 1	*Austrelaps superbus*	−9.5	2	60	13.2
sp|P60045|PA2A3_NAJSG	Acidic phospholipase A2 3 (Fragment) OS = *Naja sagittifera* PE = 1 SV = 1	*Naja sagittifera*	−2.9	1	27	10.3
sp|Q3HXY4|NGFV_PSEPO	Venom nerve growth factor OS = *Pseudechis porphyriacus* PE = 2 SV = 1	*Pseudechis porphyriacus*	−43.4	7	10	36.8
sp|Q9PUG7|PA2AH_AUSSU	Acidic phospholipase A2 S17-58 OS = *Austrelaps superbus* PE = 2 SV = 1	*Austrelaps superbus*	−10.4	2	10	14.5
sp|Q9PUG8|PA2AG_AUSSU	Acidic phospholipase A2 S16-19 OS = *Austrelaps superbus* PE = 2 SV = 1	*Austrelaps superbus*	−7.3	2	8	13.2
sp|Q6ITB0|VKT1_TROCA	Kunitz-type serine protease inhibitor carinatin-1 OS = *Tropidechis carinatus* PE = 2 SV = 1	*Tropidechis carinatus*	−9.2	2	5	37.3
sp|Q9DEZ9|NGFV_CRODU	Venom nerve growth factor OS = *Crotalus durissus terrificus* PE = 2 SV = 1	*Crotalus durissus terrificus*	−14.6	3	4	12.0
sp|G0W2I1|BDS3D_BUNGR	U-actitoxin-Bgr3d OS = *Bunodosoma granuliferum* PE = 3 SV = 1	*Bunodosoma granuliferum*	−2.8	1	3	13.9
sp|B2D0J4|VDPP4_APIME	Venom dipeptidyl peptidase 4 OS = *Apis mellifera* PE = 1 SV = 1	*Apis mellifera*	−1.6	1	3	3.5
sp|C1IC49|3NO25_WALAE	Three finger toxin W-V OS = *Walterinnesia aegyptia* PE = 1 SV = 1	*Walterinnesia aegyptia*	−2	1	2	9.3
sp|P22640|PA2H_PROMU	Basic phospholipase A2 homolog OS = *Protobothrops mucrosquamatus* PE = 1 SV = 1	*Protobothrops mucrosquamatus*	−3.5	1	2	5.7
sp|P79799|VNP32_MICCO	Natriuretic peptide Mc-NP OS = *Micrurus corallinus* PE = 2 SV = 1	*Micrurus corallinus*	−2.1	1	2	26.6
sp|P20253|PA2B9_PSEAU	Basic phospholipase A2 PA-9C OS = *Pseudechis australis* PE = 1 SV = 1	*Pseudechis australis*	−2.1	1	2	6.8

**Table A2:** Peptides identified in crude RBBV Fraction F27 Pool 2.

Accession	Protein	Hit Species	Expect	Peptide Hits	Total Peptides	Coverage
sp|Q75S48|PA2A1_BUNCA	Acidic phospholipase A2 1 OS = *Bungarus candidus* PE = 2 SV = 1	*Bungarus candidus*	−4.8	1	43	5.9
sp|P20259|PA2BB_PSEPO	Basic phospholipase A2 pseudexin B chain OS = *Pseudechis porphyriacus* PE = 1 SV = 1	*Pseudechis porphyriacus*	−34.1	5	33	35.9
sp|P20258|PA2BA_PSEPO	Basic phospholipase A2 pseudexin A chain OS = *Pseudechis porphyriacus* PE = 1 SV = 1	*Pseudechis porphyriacus*	−18.4	3	27	25.6
sp|Q3HXY4|NGFV_PSEPO	Venom nerve growth factor OS = *Pseudechis porphyriacus* PE = 2 SV = 1	*Pseudechis porphyriacus*	−62.5	9	24	45.6
sp|P20256|PA2BC_PSEAU	Basic phospholipase A2 PA-12C OS = *Pseudechis australis* PE = 1 SV = 1	*Pseudechis australis*	−11.6	2	24	17.8
sp|F8RKW5|NGFV_DRYCN	Venom nerve growth factor OS = *Drysdalia coronoides* PE = 2 SV = 1	*Drysdalia coronoides*	−21.3	4	7	12.8
sp|Q9DEZ9|NGFV_CRODU	Venom nerve growth factor OS = *Crotalus durissus terrificus* PE = 2 SV = 1	*Crotalus durissus terrificus*	−15.1	3	6	12.9
sp|Q9PUG7|PA2AH_AUSSU	Acidic phospholipase A2 S17-58 OS = *Austrelaps superbus* PE = 2 SV = 1	*Austrelaps superbus*	−11.5	2	4	14.5
sp|P20253|PA2B9_PSEAU	Basic phospholipase A2 PA-9C OS = *Pseudechis australis* PE = 1 SV = 1	*Pseudechis australis*	−7.9	2	4	11.9
sp|A8HDJ4|3S11_PSEPO	Short neurotoxin 1 OS = *Pseudechis porphyriacus* PE = 3 SV = 1	*Pseudechis porphyriacus*	−8	2	3	18.1
sp|P79799|VNP32_MICCO	Natriuretic peptide Mc-NP OS = *Micrurus corallinus* PE = 2 SV = 1	*Micrurus corallinus*	−2.2	1	3	26.6
sp|P20251|PA2A3_PSEAU	Acidic phospholipase A2 PA-3 OS = *Pseudechis australis* PE = 1 SV = 1	*Pseudechis australis*	−7.9	2	2	19.5

**Table A3:** Peptides identified in crude RBBV Fraction F27 Pool 3.

Accession	Protein	Hit Species	Expect	Peptide Hits	Total Peptides	Coverage
sp|Q75S48|PA2A1_BUNCA	Acidic phospholipase A2 1 OS = *Bungarus candidus* PE = 2 SV = 1	*Bungarus candidus*	−6.3	1	12	5.9
sp|P20259|PA2BB_PSEPO	Basic phospholipase A2 pseudexin B chain OS = *Pseudechis porphyriacus* PE = 1 SV = 1	*Pseudechis porphyriacus*	−21.4	4	10	27.4
sp|P20255|PA2BF_PSEAU	Basic phospholipase A2 PA-12A OS = *Pseudechis australis* PE = 1 SV = 1	*Pseudechis australis*	−15.5	3	10	27.1
sp|Q9PUH7|PA2A7_AUSSU	Acidic phospholipase A2 S15-109 OS = *Austrelaps superbus* PE = 2 SV = 1	*Austrelaps superbus*	−7.4	2	4	13.2
sp|Q3HXY4|NGFV_PSEPO	Venom nerve growth factor OS = *Pseudechis porphyriacus* PE = 2 SV = 1	*Pseudechis porphyriacus*	−13.5	3	3	14.2
sp|Q9PUG7|PA2AH_AUSSU	Acidic phospholipase A2 S17-58 OS = *Austrelaps superbus* PE = 2 SV = 1	*Austrelaps superbus*	−4.7	1	2	9.2
sp|Q7T2Q4|PA2A2_BUNFL	Acidic phospholipase A2 2 OS = *Bungarus flaviceps flaviceps* PE = 2 SV = 1	*Bungarus flaviceps flaviceps*	−1.6	1	2	9.5
sp|P20253|PA2B9_PSEAU	Basic phospholipase A2 PA-9C OS = *Pseudechis australis* PE = 1 SV = 1	*Pseudechis australis*	−2.1	1	2	6.8

**Table A4:** Peptides identified in crude RBBV Fraction F28 Pool 1.

Accession	Protein	Hit Species	Expect	Peptide Hits	Total Peptides	Coverage
sp|P20259|PA2BB_PSEPO	Basic phospholipase A2 pseudexin B chain OS = *Pseudechis porphyriacus* PE = 1 SV = 1	*Pseudechis porphyriacus*	−66.9	9	102	79.5
sp|P20256|PA2BC_PSEAU	Basic phospholipase A2 PA-12C OS = *Pseudechis australis* PE = 1 SV = 1	*Pseudechis australis*	−31.5	5	71	34.7
sp|P20258|PA2BA_PSEPO	Basic phospholipase A2 pseudexin A chain OS = *Pseudechis porphyriacus* PE = 1 SV = 1	*Pseudechis porphyriacus*	−25.2	4	68	29.9
sp|Q75S48|PA2A1_BUNCA	Acidic phospholipase A2 1 OS = *Bungarus candidus* PE = 2 SV = 1	*Bungarus candidus*	−5	1	14	5.9
sp|Q3HXY4|NGFV_PSEPO	Venom nerve growth factor OS = *Pseudechis porphyriacus* PE = 2 SV = 1	*Pseudechis porphyriacus*	−45	7	9	37.7
sp|Q9PUG7|PA2AH_AUSSU	Acidic phospholipase A2 S17-58 OS = *Austrelaps superbus* PE = 2 SV = 1	*Austrelaps superbus*	−11.5	2	6	14.5
sp|P20250|PA2A_PSEAU	Acidic phospholipase A2 PA-1G OS = *Pseudechis australis* PE = 1 SV = 1	*Pseudechis australis*	−14.3	3	5	35.0
sp|Q4VRI5|PA21_OXYSC	Phospholipase A2 OS1 OS = *Oxyuranus scutellatus scutellatus* PE = 1 SV = 1	*Oxyuranus scutellatus scutellatus*	−7.5	2	2	11.0
sp|P00616|PA2TG_OXYSC	Acidic phospholipase A2 homolog taipoxin gamma chain OS = *Oxyuranus scutellatus scutellatus* PE = 1 SV = 2	*Oxyuranus scutellatus scutellatus*	−8.3	2	2	14.5
sp|P49121|PA2H1_AGKCL	Basic phospholipase A2 homolog MT1 OS = *Agkistrodon contortrix laticinctus* PE = 1 SV = 1	*Agkistrodon contortrix laticinctus*	−1.6	1	2	16.1
sp|Q7LZG5|PA2_NOTSC	Phospholipase A2 II-5b (Fragment) OS = *Notechis scutatus scutatus* PE = 1 SV = 1	*Notechis scutatus scutatus*	−2.5	1	2	50.0

**Table A5:** Peptides identified in crude RBBV Fraction F28 Pool 2.

Accession	Protein	Hit Species	Expect	Peptide Hits	Total Peptides	Coverage
sp|P20259|PA2BB_PSEPO	Basic phospholipase A2 pseudexin B chain OS = *Pseudechis porphyriacus* PE = 1 SV = 1	*Pseudechis porphyriacus*	−32.8	5	14	33.3
sp|Q3HXY4|NGFV_PSEPO	Venom nerve growth factor OS = *Pseudechis porphyriacus* PE = 2 SV = 1	*Pseudechis porphyriacus*	−47.3	7	10	36.0
sp|P20258|PA2BA_PSEPO	Basic phospholipase A2 pseudexin A chain OS = *Pseudechis porphyriacus* PE = 1 SV = 1	*Pseudechis porphyriacus*	−15	3	7	25.6
sp|Q75S48|PA2A1_BUNCA	Acidic phospholipase A2 1 OS = *Bungarus candidus* PE = 2 SV = 1	*Bungarus candidus*	−4.2	1	4	5.9
sp|Q9PUG7|PA2AH_AUSSU	Acidic phospholipase A2 S17-58 OS = *Austrelaps superbus* PE = 2 SV = 1	*Austrelaps superbus*	−11	2	2	14.5
sp|E3P6P1|CYT_CRYNI	Cystatin OS = *Cryptophis nigrescens* PE = 1 SV = 1	*Cryptophis nigrescens*	−1.4	1	2	26.2
sp|P20253|PA2B9_PSEAU	Basic phospholipase A2 PA-9C OS = *Pseudechis australis* PE = 1 SV = 1	*Pseudechis australis*	−2.4	1	2	6.8

**Table A6:** Peptides identified in crude RBBV Fraction F28 Pool 3.

Accession	Protein	Hit Species	Expect	Peptide Hits	Total peptides	Coverage
sp|P20259|PA2BB_PSEPO	Basic phospholipase A2 pseudexin B chain OS = *Pseudechis porphyriacus* PE = 1 SV = 1	*Pseudechis porphyriacus*	−34.6	5	13	33.3
sp|P20258|PA2BA_PSEPO	Basic phospholipase A2 pseudexin A chain OS = *Pseudechis porphyriacus* PE = 1 SV = 1	*Pseudechis porphyriacus*	−16.7	3	10	25.6
sp|Q3HXY4|NGFV_PSEPO	Venom nerve growth factor OS = *Pseudechis porphyriacus* PE = 2 SV = 1	*Pseudechis porphyriacus*	−15	3	3	14.6
sp|Q9PUG7|PA2AH_AUSSU	Acidic phospholipase A2 S17-58 OS = *Austrelaps superbus* PE = 2 SV = 1	*Austrelaps superbus*	−5.2	1	3	9.2

**Table A7:** Peptides identified in crude RBBV Fraction F29 Pool 1.

Accession	Protein	Hit Species	Expect	Peptide Hits	Total Peptides	Coverage
sp|P20259|PA2BB_PSEPO	Basic phospholipase A2 pseudexin B chain OS = *Pseudechis porphyriacus* PE = 1 SV = 1	*Pseudechis porphyriacus*	−89.4	11	168	89.7
sp|Q3HXY4|NGFV_PSEPO	Venom nerve growth factor OS = *Pseudechis porphyriacus* PE = 2 SV = 1	*Pseudechis porphyriacus*	−38.8	6	6	31.4
sp|P0DN62|TF4_TERSU	Teretoxin Tsu15.4 OS = *Terebra subulata* PE = 3 SV = 1	*Terebra subulata*	−2.1	1	4	44.2
sp|Q9DEZ9|NGFV_CRODU	Venom nerve growth factor OS = *Crotalus durissus terrificus* PE = 2 SV = 1	*Crotalus durissus terrificus*	−15.1	3	3	12.9
sp|P79799|VNP32_MICCO	Natriuretic peptide Mc-NP OS = *Micrurus corallinus* PE = 2 SV = 1	*Micrurus corallinus*	−2.3	1	3	26.6

**Table A8:** Peptides identified in crude RBBV Fraction F29 Pool 2.

Accession	Protein	Hit Species	Expect	Peptide Hits	Total Peptides	Coverage
sp|P20259|PA2BB_PSEPO	Basic phospholipase A2 pseudexin B chain OS = *Pseudechis porphyriacus* PE = 1 SV = 1	*Pseudechis porphyriacus*	−59.6	8	21	47.9
sp|Q3HXY4|NGFV_PSEPO	Venom nerve growth factor OS = *Pseudechis porphyriacus* PE = 2 SV = 1	*Pseudechis porphyriacus*	−47.4	7	12	39.7
sp|P04056|PA2BB_PSEAU	Basic phospholipase A2 PA-11 OS = *Pseudechis australis* PE = 1 SV = 1	*Pseudechis australis*	−9	2	6	17.8
sp|Q9PUH7|PA2A7_AUSSU	Acidic phospholipase A2 S15-109 OS = *Austrelaps superbus* PE = 2 SV = 1	*Austrelaps superbus*	−8.3	2	5	13.2
sp|P59069|PA2SC_AUSSU	Phospholipase A2 superbin c (Fragment) OS = *Austrelaps superbus* PE = 1 SV = 1	*Austrelaps superbus*	−7.6	2	2	47.8
sp|Q9BPC1|O262_CONTS	Conotoxin TsMEKL-P012 OS = *Conus tessulatus* PE = 2 SV = 1	*Conus tessulatus*	−1.5	1	2	25.6

**Table A9:** Peptides identified in crude RBBV Fraction F29 Pool 3.

sp|P20259|PA2BB_PSEPO	Basic phospholipase A2 pseudexin B chain OS = *Pseudechis porphyriacus* PE = 1 SV = 1	*Pseudechis porphyriacus*	−57.2	8	16	47.9
sp|P20250|PA2A_PSEAU	Acidic phospholipase A2 PA-1G OS = *Pseudechis australis* PE = 1 SV = 1	*Pseudechis australis*	−9.5	2	3	22.2
sp|Q9PUH7|PA2A7_AUSSU	Acidic phospholipase A2 S15-109 OS = *Austrelaps superbus* PE = 2 SV = 1	*Austrelaps superbus*	−8.3	2	3	13.2
sp|Q45Z27|PA2A4_TROCA	Acidic phospholipase A2 4 OS = *Tropidechis carinatus* PE = 2 SV = 1	*Tropidechis carinatus*	−8	2	3	13.2
	(1)					

**Table A10:** Peptides identified in crude RBBV Fraction F35 Pool 1.

Accession	Protein	Hit Species	Expect	Peptide Hits	Total Peptides	Coverage
sp|P85061|PA23_ACASS	Phospholipase A2 acanmyotoxin-3 (Fragment) OS = *Acanthophis* sp. (strain Seram) PE = 1 SV = 1	*Acanthophis* sp. (strain Seram)	−3.1	1	27	33.3
sp|Q9PUH4|PA2AA_AUSSU	Acidic phospholipase A2 S5-32M OS = *Austrelaps superbus* PE = 2 SV = 1	*Austrelaps superbus*	−24.1	4	25	26.0
sp|Q9I837|PA2BG_LATSE	Basic phospholipase A2 GL1-1 OS = *Laticauda semifasciata* PE = 2 SV = 1	*Laticauda semifasciata*	−8.2	2	12	12.4
sp|Q3HXY4|NGFV_PSEPO	Venom nerve growth factor OS = *Pseudechis porphyriacus* PE = 2 SV = 1	*Pseudechis porphyriacus*	−50.7	7	10	36.8
sp|P20259|PA2BB_PSEPO	Basic phospholipase A2 pseudexin B chain OS = *Pseudechis porphyriacus* PE = 1 SV = 1	*Pseudechis porphyriacus*	−38.3	6	8	47.0
sp|Q3HXX4|NGFV5_TROCA	Venom nerve growth factor 5 OS = *Tropidechis carinatus* PE = 2 SV = 1	*Tropidechis carinatus*	−23.9	4	6	17.7
sp|Q3HXY3|NGFV1_PSEAU	Venom nerve growth factor 1 OS = *Pseudechis australis* PE = 2 SV = 1	*Pseudechis australis*	−23.3	4	6	14.5
sp|Q9DEZ9|NGFV_CRODU	Venom nerve growth factor OS = *Crotalus durissus terrificus* PE = 2 SV = 1	*Crotalus durissus terrificus*	−8.7	2	4	9.1
sp|P20258|PA2BA_PSEPO	Basic phospholipase A2 pseudexin A chain OS = *Pseudechis porphyriacus* PE = 1 SV = 1	*Pseudechis porphyriacus*	−13.8	3	3	25.6
sp|P00614|PA2TA_OXYSC	Basic phospholipase A2 taipoxin alpha chain OS = *Oxyuranus scutellatus scutellatus* PE = 1 SV = 1	*Oxyuranus scutellatus scutellatus*	−7.5	2	2	21.0
sp|Q9BPC1|O262_CONTS	Conotoxin TsMEKL-P012 OS = *Conus tessulatus* PE = 2 SV = 1	*Conus tessulatus*	−1.7	1	2	25.6
